# Observations on the Eau Medicinale D'Husson

**Published:** 1810-11

**Authors:** Joshua Sylvester


### Medical and Physical Journal.

VOL. XXIV.] NOVEMBER, 1810. [NO. 141.

Printed for R. PHILLIPS, by E. Hemsted, Great New Street, Fetter Lane, London.

### *To the Editors of the Medical and Physical Journal.*

"We appear astonished when we see the multitude led away by sounds; but if sounds work miracles, it is always upon ignorance: the influence of names is in exact proportion to the want of knowledge."—PALEY.

GENTLEMEN,

ABOUT thirty years ago, there came from France to this Metropolis a *great* Doctor named Le Fevre; and he was of his time, "the grand composer of all human woes." He soared much higher than D'Husson, the inventor only of the Eau Medicinale, not the propagator, the English have done that for him. One hundred guineas were paid down upon the nail to Le Fevre, and then he administered his medicine himself. He, from his high price, only embraced the richest freeholders in England, and his patients were found in the grandest squares in London. He was not heard of in Piccadilly, as Piccadilly then was, nor scarcely was there a patient rich enough for him in Soho Square. Monsieur Le Fevre did his business well. There was no loitering nor lingering in his plan. He was here only for a season. There happened to be in the history of credulity a rich commoner of the name of Brachenbury, of the family of Brachenbury, Governor of the Tower in Richard the Third's time, a gentleman of Cambridgeshire, who took Le Fevre's medicine, and who died under the process of it as administered by the Doctor in person; and his son Charles succeeded to his estates at an early part of his life, before he had left his rotine degrees at the University of Cambridge. This incident, so very flattering to the tempting prosperity of youth, became an animated subject for their conversation; and at their clubs and their associations, they never failed to drink the health of *Doctor Le Fevre*, trusting in their turns to the fortunate credulity of their own gouty fathers. But no sooner was Doctor Le Fevre smoaked, than he decamped, promising to return the next

 season.

season. That season has never returned, consequently we have heard no more of the Doctor Le Fevre.

He has been said to have realised 50,000*l.* by his prosperous play on English credulity. I have said English credulity, not British, because I really think the northern Learned could not have been so easily amused. But if the medicine and the terms of D'Husson be more pettifogging than that of Le Fevre, yet all about D'Husson is more dangerous to society. D'Husson's medicine is effective, and its circulation is much more general. It is bought by all those who love quackery, and can and do feel a pride in setting up for themselves. If they should happen to be too mean in their nature, and which is often the case, to swallow the proportions D'Husson has directed, rather than not be said to take his medicine upon their own account, they will take that in drop by drop which he has prescribed by spoonfuls. Hence it is that we do not hear of all the mischief, or all the good, as a Cambridge youth would have called it thirty years ago, which it is capable of doing.

Mr. Hughes, the late treasurer of Covent-garden Theatre, swallowed the doses of the Eau Medicinale D'Husson, by the instructions of the vender. He made but two bites of this deleterious cherry. It brought upon him the combined actions of an emetic, a cathartic, a cold sweat and syncopé. Incessant vomitings, purgings, and cold sweats, with privation of all strength, soon too fatally convinced Mr. Hughes of the power possessed by this grand composer. D'Husson has not confined the power of this grand composer to the cure of the gout. Read his bill, and you will find that the praise he embraces of it is much more liberal, it includes all the certain virtues of all the quack medicines, in all the shops of patent quackery in the first streets of this metropolis. Read D'Husson's bill of the attributes of the Eau Medicinale D'Husson.

Who was this Husson? An half-pay officer, it is said. Half-pay officers in France are not to be compared with those in England. Few English officers depend only upon half-pay, and fewer from spirit submit to the shifts for subsistence upon quackery. This hungry medicine has lingered as an invention for years; and, as a prophet is no prophet in his own country, it would have undoubtedly turned out an abortion, if some generous Englishman had not taken compassion from sympathy on its ricketty imbecillity.

What is the composition of the Eau Medicinale D'Husson?

And if the composition cannot be otherwise declared,

What is the comparative action of this Eau Medicinale D'Husson?

This is the point I mean to investigate.———

A colonel

A colonel in the army, lately from Portugal, put one of those curious looking bottles into my hands. I tasted the content, and I recollected something like it. Finding that I recollected a similar taste, I bought one of them. I walked about all the morning tasting it; my confirmation grew more and more strengthened, that the efficient material of the Eau Medicinale was Tobacco. Its taste and action in every respect have confirmed it.

After having tasted it on through the whole of the morning, I smoaked a pipe of tobacco, and I found the effect still more confirmed. I then sweetened some white wine, and added to it a little of the infusion of tobacco, with a small quantity of infusion of Columbo root. I gave a boy a taste of both; he affirmed that the taste of both was alike. I gave a girl also a taste of both, and she affirmed it also. The boy and girl were not known to each other. I judged this to be the best method of ascertaining the fact, by thus trying it upon an unadulterated taste. It is well known that water-drinkers are the best judges of the different flavour of wine.

I think D'Husson, by the turbidity of this coarse mixture, gives into it nothing of tobacco but the infusion. An half-pay officer who turns quack has seldom so much of talent as he has of misery; and if we say, that necessity is the mother of invention, yet this invention belongs not to genius, but is confined to the wants of necessity. The simple talent of a quack would not lead him to prepare an extract, or an oil. Those who do not, ought to know, that the oil and extract of tobacco are amongst the strongest and most deleterious poisons of the vegetable kingdom. The Abbé Fontana, the most intelligent philosopher of this age, has said it and proved it by his own experiments.

I shall for the present proceed no farther in my investigation. But I cannot close the subject without advising the curious sceptic who doubts the facts I have advanced, not to depend upon the mixture I gave, if it be put into any bottle. No. It must be put into one of D'Husson's vender's bottles, and it must be wrapped all over with the magical mummery of paper and printing, and sealed like his, and then, and then only let the comparison of taste and effect be made.

On the first importation of tobacco into England, it was then recommended by the interested for its various powers of curing diseases and restoring health. The properties and the name of the herb were then avowed; whereas at this day its familiarity would not admit of the secret being imparted; because, by the name and property of the herb being known, the charm of the novelty would have been dissolved, the vulgar antidote would have its salutary qualities disowned, and the vender exposed.

But till now the Tobacco-herb has been confined to the pipe, the mouth, and the snuff-box, except occasionally and cautiously it has been given in clysters, when its effect is very powerful indeed, and brings on cold sweats, syncopé, and sometimes, with all our caution, even death. It has not been permitted in any quantity to pass into the stomach knowingly for any good.

The quackery of tobacco has never been from time to time unnoticed. Every age has had its attention called to it. Even the poets of the brightest fame have been celebrated under the fumes of this deleterious and loathsome weed.* And as far back as in the reign of James the First, a Satyrical Poem was written upon it, dedicated to Sir George Villers, by Joshua Sylvester, and from which the following are extracts.

The Poem is entitled,

TOBACCO BATTERED, or at least wise
Ouer-loue so loathsome Vanitie.
by
A Volley of holy Shot
Thundered
From Mount Helicon.

---

It must not be forgotten, that the application of Tobacco in this Poem to the human constitution, is confined to chewing, smoaking, and snuffing.

FIRST.

*The Quackery on the Vertues of Tobacco.*

" 'Twere therefore better somewhat else to seek
Than rest in this, so worthie of *dislike;*
Sith curing thus one small infirmity,
It doth create a greater malady,
When thereby freed (perhaps) from *Gout* we fall
In bondage of this *custome* capitall.
For they that *physicks* to a *custome* bring,
Bring their *disease* too, to accustoming.
Perpetuall *physicke* must of force imply
Perpetuall *sicknesse*; or deep foolerie
Compos'd of *anticke* and of phrantick too:
For where's no *sickness*, what should med'cine doo?"

---

* See Oxford Sausage, by Browne.

SECOND.

SECOND.

*On the loathsom Offence in Society, of those who accustom themselves to Tobacco.*

" Offend their friends with a most *un-respect*,
Offend their wives, and children with neglect;
Offend the eyes, with foule and loathsom sprawlings;
Offend the nose with filthy fumes exhalings;
Offend the ears with loud lewd *execrations*;
Offend the mouth with ougly excreations;
Offend the *sense*, with stupefying sense;
Offend the weake, to follow their offense:
Offend the body, and offend the minde;
Offend the *conscience* in a fearfull kinde:
Of all EXCUSE (saue fashion, custome, will),
In so apparent, proued, granted, *ill*,
Woe, woe to them by whom *offences* come,
So scandalous to all our *Christendome*.

THIRD.

*On the Characters of those who practised and promoted the Use of it.*

" Oft have I seen fooles of all sorts frequent it,
Fooles of all size, fooles of all sexes hent it;
Fooles of all colours, fooles of all complexions,
Fooles of all fashions, fooles of all affections,
Fooles natural, fooles artificial,
Fooles RICH and poor, young fooles, old fooles and all,
Whome foole I pitied, for their wilfull folly;
Supposing some discreetly wise (for holy)
Could be entangled with so fond a thing."

And thus you have had the real and best opinion I have to offer upon l'Eau Medicinale D'Husson.

I am, with all respect,
The great, great, great grandson of
JOSHUA SYLVESTER.

